# Chitosan-Oligosaccharide-Bearing Biphasic Calcium Phosphate Bone Cement: Preparation and Angiogenic Activity In Vitro

**DOI:** 10.3390/molecules30112286

**Published:** 2025-05-23

**Authors:** Jianshen Liu, Xinghua Guo, Qishi Che, Zhengquan Su

**Affiliations:** 1 Guangdong Engineering Research Center of Natural Products and New Drugs, Guangdong Provincial University Engineering Technology Research Center of Natural Products and Drugs, Guangdong Pharmaceutical University, Guangzhou 510006, China; 13128483074@163.com (J.L.); guoxinghua_chujie@163.com (X.G.); 2 Guangdong Metabolic Disease Research Center of Integrated Chinese and Western Medicine, Key Laboratory of Glucolipid Metabolic Disorder, Ministry of Education of China, Guangdong TCM Key Laboratory for Metabolic Diseases, Guangdong Pharmaceutical University, Guangzhou 510006, China; 3 Guangzhou Rainhome Pharm & Tech Co., Ltd., Science City, Guangzhou 510663, China; cheqishi@rhkj.com.cn

**Keywords:** physical performance optimization, calcium phosphate cement, chitosan oligosaccharide, angiogenesis in vitro

## Abstract

Although calcium phosphate bone cement has some advantages (it is easy to form, self-curing, and does not produce heat), some disadvantages remain that limit its clinical application. Therefore, the question of how we can modify CPC and further improve the various properties of calcium phosphate bone cement is a current research hotspot. In this paper, the preparation conditions and technology of biphasic calcium phosphate (BCP) were optimized; chitosan oligosaccharide (COSM) with MW ≤ 3000 Da was added to the optimal formulation of biphasic calcium phosphate cement particles, and its physical and chemical properties were characterized. The results showed that BCP bone cement carrier for clinical operations was successfully constructed by the high-temperature solid-state reaction method, and COSM-BCP bone cement particles were obtained by loading COSM drugs with an angiogenesis effect. Its formula is biphasic calcium phosphate powder with the molar ratio of α-TCP/β-TCP of 1. The curing time of the prepared BCP particles is 24 ± 1 min, the compressive strength is 29.58 ± 1.89 MPa, and the porosity reaches 52.09%. The loaded COSM can be released continuously and stably in vitro, and has the effect of promoting angiogenesis. The safety evaluation of COSM-BCP bone cement particles and the preliminary pharmacodynamic study of its angiogenesis in vitro provide a promising clinical application basis for the development of drug-loaded biological bone substitute materials.

## 1. Introduction

Bones are important organs in the human body, providing structural support for exercise and protecting other organs in the body. However, when serious trauma, malignant tumor, infection, etc., are encountered, the dynamic balance of bone repair tilts in a bad direction [1,2]. For example, when diabetes is encountered, it can affect the normal functioning of tissues, organs, and even bones, thereby damaging bone quality and leading to osteoporosis, which increases the risk of fractures [3,4]. In the United States alone, about 500,000 patients receive bone transplants every year, with international rates being even higher [5]. Autologous bone transplantation is regarded as the “gold standard” due to its minimal risk of immune rejection and infection during tissue regeneration. However, it requires harvesting bone material from the patient, often resulting in prolonged postoperative pain [6]. The use of allogenic or xenogenic bone requires careful evaluation. While these materials exhibit excellent bone conductivity and structural similarity, their application is often limited by risks such as inflammation, immune rejection, and potential transmission of infectious diseases. These factors contribute to a higher failure rate for allografts and xenografts compared to autologous bone transplantation [7]. Based on the characteristics of natural bone components, biological bone substitute materials have been developed, offering safety, non-toxicity, and immunity from rejection. These materials are relatively inexpensive and readily available, making their use increasingly widespread in treatment applications.

Bone is a highly vascularized tissue, and inadequate blood vessel supply can significantly reduce bone formation potential and lead to a decrease in bone mass. Research has revealed a strong spatiotemporal correlation between osteogenesis and angiogenesis, a phenomenon referred to as “angiogenesis–osteogenesis coupling” [8,9]. In addition, angiogenesis leads to the presence of cell types and growth factors involved in bone tissue repair. In the process of bone healing, callus with rich blood vessels is formed, which provides cells essential for bone regeneration at fracture sites [10,11]. An angiogenesis strategy can promote bone absorption and formation, which are widely recognized issues in bone tissue engineering. In a study by Mirali et al. [12], scaffolds promoting angiogenesis were found to be more effective, faster, and more comprehensive than bone xenografts and collagen membranes alone. The newly formed blood vessels provided essential nutrients, oxygen, growth factors, and hormones necessary for bone tissue regeneration. In a word, the supply of blood vessels in tissue engineering implants may have a positive impact on the process of bone integration and bone defect repair and should also be considered when preparing bone tissue repair materials.

Inorganic bioceramics used to treat bone defects have been widely studied because of their good biocompatibility, biodegradability, bone conductivity, and osteoinductivity [13]. Because their chemical composition is closely related to the inorganic components of natural bone tissue, these materials typically exhibit excellent biocompatibility, especially when compared to other bone repair materials such as titanium and stainless steel [14]. Since the early 1980s, according to the work done by LeGeros and Brown [15], calcium phosphate has also been used to produce cement that self-sets at body temperature. These bone cements are usually set in bioabsorbable phase, allowing them to be gradually replaced by new bone tissue. Tricalcium phosphate (TCP, Ca_3_(PO_4_)_2_), one of the most extensively studied calcium phosphates alongside hydroxyapatite, is a calcium phosphate with a calcium-to-phosphorus ratio of 1.5, which exists in two distinct phases: alpha and beta. β-TCP has a more stable structure and a higher biodegradation rate compared to α-TCP, making it more commonly used for bone regeneration [16]. β-TCP is less stable than HAP, but it degrades more quickly and has higher solubility. In addition, it has a high absorption rate and is widely used to increase biocompatibility [17]. Thus, in order to make use of the characteristics of TCP and HAP at the same time, biphasic materials were developed. Biphasic or multiphase calcium phosphate exists in the form of non-separation, because each component is evenly and closely mixed at submicron level [18]. The biphasic form of calcium phosphate was first developed in 1986 as a mixture of hydroxyapatite and β-tricalcium phosphate [19]. These biphasic calcium phosphates usually combine two more incompatible calcium phosphates, which are mainly evaluated in terms of biological activity, bioabsorbability and osteoinductivity [20]. Biphasic calcium phosphate has been used as bone graft, bone substitute, and dental material [21]. Ramay et al. [22] developed a biodegradable, porous nanocomposite scaffold composed of a β-TCP matrix and HAP nanofibers. The combination of hydroxyapatite and beta-tricalcium phosphate can stimulate osteogenic differentiation of mesenchymal stem cells, enhance cell adhesion, support growth factor attachment, and improve mechanical properties, making it widely applicable in various fields [23,24].

As a bone repair material, drug-loaded bone cement is widely used in the treatment of bone defects and bone diseases. Its main advantage is that it can achieve local, slow release of drugs and reduce systemic side effects. Yung et al. loaded antibiotics such as vancomycin into bone cement for the treatment of infectious bone defects and found that they could effectively inhibit local infection and promote bone healing [25]. The long-term complications of current clinical treatment prompt people to look for safer substitutes. Pharmacological studies indicate that natural pharmaceutical compounds play a significant role in bone regeneration. For instance, icariin promotes bone formation by activating the ER-α-Wnt/β-catenin signaling pathway [26]. Chitosan oligosaccharides (COSs) are the degradation products of chitosan, which are low polymers formed by β-(1→4)-glycosidic bonds between glucosamine and N-acetylglucosamine. It has been found that chitosan oligosaccharides participate in anti-inflammatory, antibacterial, and anti-tumor reactions [27], and they also attract attention in the field of bone repair due to their good biological activity. Studies have shown that chito-oligosaccharides can not only promote angiogenesis but also promote the proliferation and differentiation of osteoblasts and accelerate the process of bone mineralization. Huang et al. found that COSs can create an immunomodulatory microenvironment, proteins associated with osteogenesis and angiogenesis, as well as enhancing osteogenic and angiogenic differentiation, promoting the osteogenic potential of bone mesenchymal stem cells (BMSCs) and stimulating the vascular activation of human umbilical vein endothelial cells (HUVECs) [28]. Selvaraj et al. conducted experiments using mouse bone marrow mesenchymal stem cells (MSC) and zebrafish models, and found that COSs can promote osteoblast differentiation and anti-osteoporosis activity [29].

Although calcium phosphate cement has the advantages of easy molding, self-curing, and no heat generation, there are still some problems such as insufficient toughness and low mechanical strength which restrict the development and further popularization and application of this cement. In this study, starting with the synthesis of biphasic calcium phosphate with different content ratios, the properties of calcium phosphate bone cement were optimized using a multi-component system; the mechanical properties and drug-release properties of the bone cement were improved by adding drug-loaded COSM. Finally, COSM was loaded to study its effect on promoting bone angiogenesis. The purpose of this work is to provide an effective strategy for promoting bone tissue growth by using chitosan oligosaccharide in bone tissue engineering.

## 2. Results

### 2.1. Material Properties

#### 2.1.1. XRD Patterns

**Figure 1:**
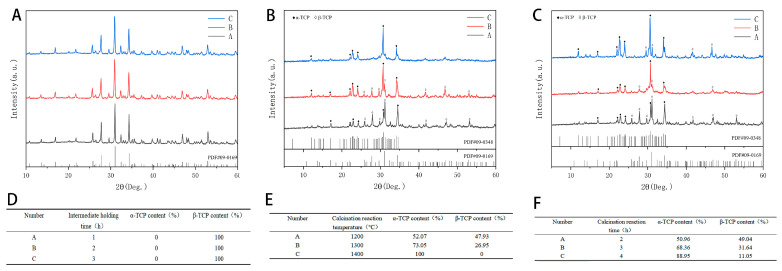
XRD pattern and physicochemical data. (**A**) XRD diagram of tricalcium phosphate powder synthesized with different intermediate holding times. (**B**) XRD diagram of tricalcium phosphate powder synthesized with different calcination reaction temperatures. (**C**) XRD diagram of tricalcium phosphate powder synthesized with different calcination reaction times. (**D**) Content ratio of different crystalline tricalcium phosphate synthesized with different intermediate holding times. (**E**) Content ratio of different crystalline tricalcium phosphate synthesized with different calcination reaction temperatures. (**F**) Content ratio of different crystalline tricalcium phosphate synthesized with different calcination reaction times.

Here, we explore the effect of incubation time at 900 °C for 1 h, 2 h, and 3 h on the synthesis of β-TCP. Compared with the β-TCP standard card PDF#09-0169, the position and intensity of the diffraction peaks of β-TCP synthesized in three holding times are consistent, and there are no other peaks (Figure 1A). Jade 6.5 was used to determine and analyze the content, and the composition ratio of phases in calcined products at different intermediate holding times was obtained. The β-TCP content of calcined products could reach 100% (Figure 1D). To sum up, when the temperature reaches 900 °C, it can be completely converted into β-TCP after calcination for 1 h; no other products appear after prolonged calcination time, and the precursor is relatively stable. In order to save energy and calcination time efficiency, generally, a 1 h intermediate holding time can be selected for subsequent calcination treatment.

Here, we explore the influence of different calcination reaction temperatures on the synthesized TCP crystal phase for the same holding time (4 h) (Figure 1B). Compared with the α-TCP standard card PDF#09-0348 and β-TCP standard card PDF#09-0169, the α-TCP synthesized at three calcination reaction temperatures has the same diffraction peak position and intensity, and there are no other peaks. Jade 6.5 was used to determine and analyze its content, and the phase composition ratio of the product at different calcination reaction temperatures was obtained. With the increase in calcination reaction temperature, the composition ratio of α-TCP also increases gradually (Figure 1E). When the calcination temperature reached 1400 °C and the reaction time was 4 h, the pure β-TCP was completely converted into α-TCP. This reaction parameter can be used as a reference for producing a pure α-TCP phase. In addition, no other products were produced except α-TCP and β-TCP at the three calcination reaction temperatures.

Here, we explore the influence of different calcination reaction times on the synthesized TCP crystal phase at the same calcination reaction temperature (1300 °C) (Figure 1C). α-TCP and β-TCP synthesized at three calcination reaction times all have strong diffraction peaks at their top eight peaks. Similarly, compared with PDF#09-0348 and PDF#09-0169, the positions and intensities of diffraction peaks are relatively consistent, and there are no other peaks. According to the content analysis results of Jade 6.5, the longer the calcination reaction time, the higher the α-TCP content (Figure 1F). In addition, in the process of transforming β-TCP into α-TCP, no other phase was produced. Therefore, in this study, through curing time and compressive strength, the primary calcination temperature of 900 °C, holding for 1 h, and the secondary calcination temperature of 1300 °C, holding for 2 h, were selected; the powders for subsequent investigations were fired on this basis.

#### 2.1.2. SEM Results

**Figure 2:**
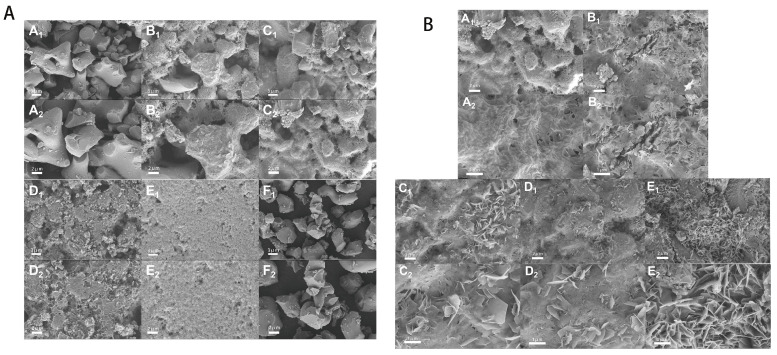
(**A**(**A_1_**,**A_2_**)) SEM images of β-TCP hydration, with magnifications of 3000 times and 5000 times, respectively; (**A**(**B_1_**,**B_2_**)) SEM images of hydration of TCP powder with an α/β-TCP ratio of 1:3, with magnifications of 3000 times and 5000 times, respectively; (**A**(**C_1_**,**C_2_**)) SEM images of hydration of TCP powder with an α/β-TCP ratio of 1, with magnifications of 3000 times and 5000 times, respectively; (**A**(**D_1_**,**D_2_**)) SEM images of hydration of TCP powder with an α/β-TCP ratio of 3:1, with magnifications of 3000 times and 5000 times, respectively; (**A**(**E_1_**,**E_2_**)) SEM images of hydration of TCP powder with an α/β-TCP ratio of 1:0, with magnifications of 3000 times and 5000 times, respectively; (**A**(**F_1_**,**F_2_**)) SEM image of TCP powder with α-/β-TCP ratio of 1, with magnifications of 3000 times and 5000 times, respectively; (**B**(**A_1_**–**E_2_**)) SEM images of bone cement loaded with 0 wt%, 1 wt%, 2 wt%,5 wt% and 10 wt% COSM, with magnifications of 5000 times and 15,000 times, respectively.

Here, we discuss the morphology of hydration products with different molar ratios of α-TCP/β-TCP and the microscopic morphology of the powder with a molar ratio of 1 (Figure 2A). As shown in Figure 2A(F_1_,F_2_), the morphology of unhydrated biphasic calcium phosphate powder is fine, comprising irregular particles with an average particle size of about 50 μm, and there is no obvious agglomeration phenomenon. As shown in Figure 2A(A_1_,A_2_), CPC particles were formed by mixing pure β-TCP. It can be seen that combination between particles is compact and dense. Because β-TCP does not participate in hydration, the curing liquid only shapes it; its pore size is relatively large and porosity is high. In Figure 2A(B_1_–E_2_), with the increase in the α-TCP/β-TCP molar ratio, the surface morphology of the CPC hydration products changed from a granular structure to a scaly structure. These scaly products should be the CDHA phase, which is the product of α-TCP hydration. Calcium-deficient hydroxyapatite grows on the surface of biphasic α, β-tricalcium phosphate powder in the form of scales, which will also prevent α-TCP from further hydration. With the increase in the molar ratio of α-TCP/β-TCP, the formed CDHA increases, which makes the pores of CPC closed and smaller, and the porosity gradually decreases, which further improves the mechanical properties. However, pore size and porosity that are too small are not conducive to bone repair, so it is necessary to find a balance between pore structure and mechanical strength.

Figure 2B shows a microscopic diagram of biphasic calcium phosphate cement (α-TCP/β-TCP molar ratio is 1) loaded with different contents of COSM. As can be seen from Figure 2B(A_1_,A_2_), CPC particles without COSM are covered with a layer of hydrated CDHA phase, and irregular pores are formed due to the existence of β-TCP; the CDHA covering the surface further hinders the hydration of α-TCP, thus retaining good porosity. As shown in Figure 2B(B_1_–E_2_), with the addition of COSM, the surface morphology of CPC hydration products presents a scale-like structure, and with the increase in COSM content, its surface pores are closed and reduced; accordingly, it can be guessed that its compressive strength is improved. The reason for this change may be that the addition of COSM delayed the curing process of CPC and made it possible for it to fully hydrate into CDHA.

#### 2.1.3. MIP Results

**Figure 3:**
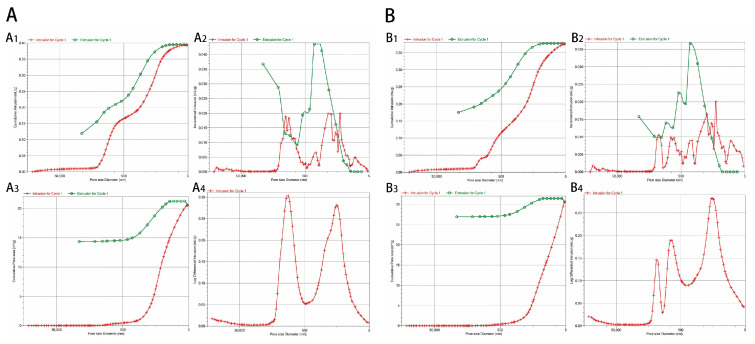
Relationship diagram between cumulative mercury intake and pore size (**A_1_**,**B_1_**); diagram of the relationship between incremental mercury intake and pore size (**A_2_**,**B_2_**); diagram of the relationship between total pore area and pore size (**A_3_**), and the relationship between logarithmic differential mercury intake and pore size of biphasic calcium phosphate cement (**A_4_**); diagram of the relationship between total pore area and pore size (**B_3_**), and the relationship between logarithmic differential mercury intake and pore size of biphasic calcium phosphate cement loaded with COSM (**B_4_**).

The relevant analysis results of MIP detection after the hydration and curing of biphasic calcium phosphate cement for 3 days are shown in Figure 3A. The porosity calculated using the Washburn equation is 52.09%, which meets the requirements of bone tissue engineering. In addition, its average pore diameter 4V/A is 76.59 nm, its total pore volume is 0.3938 mL/g, and its total pore area is 20.568 m^2^/g. The Washburn equation is calculated as follows:

(1) $$L^{2}=\frac{\gamma \cos\theta }{2\eta }t$$

The relevant analysis results for the MIP detection of biphasic calcium phosphate cement with 5 wt% COSM after hydration and curing for 3 days are shown in Figure 3B. According to the Washburn equation, its porosity is 50.18%, its average pore diameter is 49.16 nm, its total pore volume is 0.3761 mL/g, and its total pore area is 30.597 m^2^/g. Compared with biphasic calcium phosphate cement without COSM, its porosity decreased slightly, and the total pore volume did not change much, but the average pore diameter decreased by 1/3. Combined with the analysis of the scanning electron microscope results, after adding COSM, the hydration process was prolonged, and CDHA increased, which had a certain covering effect on the cement. In terms of total pore volume and total pore area, COSM-BCP lacks macropores and through-holes, which is not conducive to vascular growth and degradation compared with blank BCP.

### 2.2. Analysis of Curing Time and Compressive Strength Results

**Figure 4:**
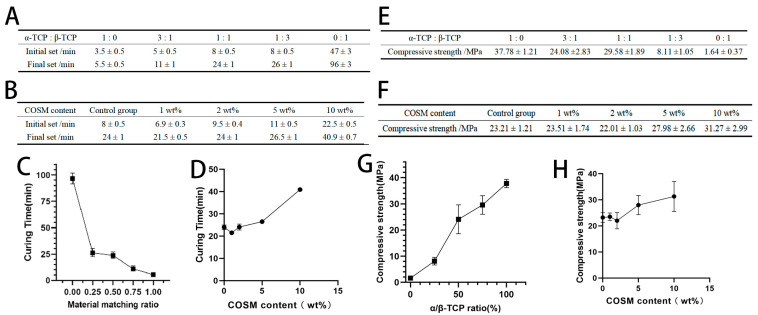
Analysis of curing time and compressive strength results. (**A**) Comparison of initial setting and final setting times of different crystal TCP content ratios ($\bar{x}$ ± s, *n* = 3). (**B**) Comparison of initial setting and final setting times of bone cement loaded with different contents of COSM ($\bar{x}$ ± s, *n* = 3). (**C**) Comparison of curing times of TCP content ratios of different crystal forms (*n* = 3). (**D**) Comparison of curing times of bone cement loaded with different contents of COSM (*n* = 3). (**E**) Compressive strengths of different crystal TCP content ratios ($\bar{x}$ ± s, *n* = 5). (**F**) Comparison of compressive strengths of bone cement loaded with different contents of COSM ($\bar{x}$ ± s, *n* = 5). (**G**) Comparison of compressive strengths of different crystal TCP content ratios (*n* = 5). (**H**) Comparison of compressive strengths of bone cement loaded with different contents of COSM (*n* = 5).

As shown in Figure 4A,C, the hydration reaction of pure α-TCP to generate CDHA makes the setting time extremely short. With the addition of β-TCP, the final setting time of BCP bone cement continues to increase. Because β-TCP does not participate in hydration, the α-TCP powder that can undergo hydration reaction decreases, the actual liquid–solid ratio of the system increases, and the solidification time is prolonged. The curing principle of pure β-TCP involves the volatilization of excess water, thus reaching a certain strength, so the final setting time is greatly increased. Figure 4B,D show the curing time comparison of BCP loaded with different contents of COSM. When the content of COSM is 1 wt%, the initial setting and final setting time of BCP is slightly reduced compared with that of BCP without COSM; this may be because adding an appropriate amount of COSM can accelerate the dissolution rate of α-TCP, leading it to quickly form a stable structure and shorten the curing time. However, with the increase in COSM content, the curing time of COSM-BCP also increased with the increase in COSM content. Excessive COSM slows down the process of liquid diffusion, and the formation time of the CDHA shell is prolonged, which makes it impossible to form a stable structure; then, the curing time is prolonged [14].

The compressive strength of pure β-TCP bone cement is the lowest, which is 1.64 ± 0.37 MPa (Figure 4E,G). With the increase in α-tricalcium phosphate content, the compressive strength of biphasic calcium phosphate cement generally showed a trend of great increase, and the compressive strength of pure α-TCP cement reached the maximum, which was 37.78 ± 1.21 MPa. A comparison between the different compressive strengths of BCP loaded with different contents of COSM is shown in Figure 4F,H. When the content of COSM is 1 wt% and 2 wt%, the compressive strength of BCP without COSM has no obvious change. However, when COSM content is 5 wt% and 10 wt%, its compressive strength is obviously higher than that of other groups, and the compressive strength with large COSM content is higher. According to the MIP analysis results, the addition of COSM can reduce the pore size and porosity, but it leads to the enhancement of the compressive strength of COSM-BCP. In addition, the increase in COSM content is positively correlated with the curing time of BCP, which is beneficial in strengthening the hydration degree and the precipitation degree of CDHA crystals.

### 2.3. In Vitro Drug Release Results

**Figure 5:**
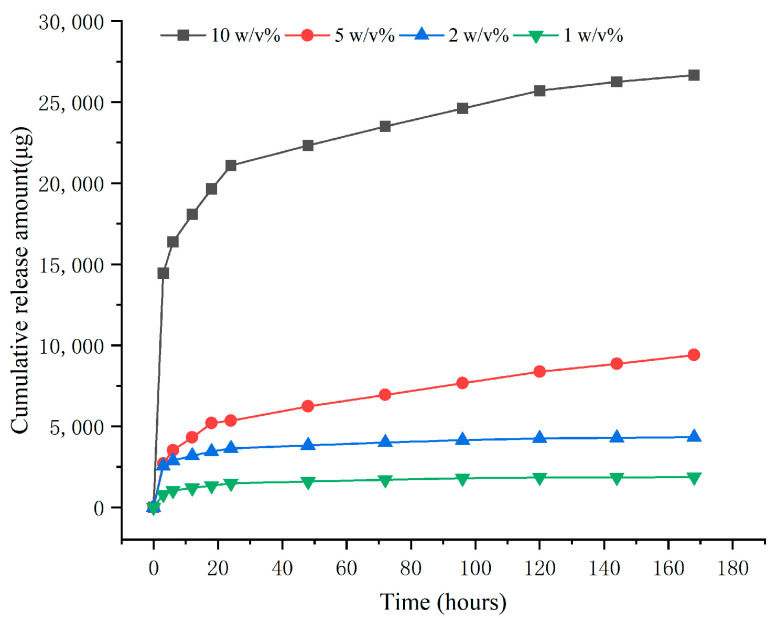
In vitro drug-release curve of COSM-BCP loaded with different contents of COSM.

The in vitro release curve of BCP loaded with different contents of COSM is shown in Figure 5. COSM with different contents can be released stably and continuously in the drug-loaded system. The BCP of 10 wt% COSM showed the initial burst release of COSM, and it was slowly released after 12 h. With the decrease in COSM content, the release of COSM-BCP in each group became more sustained and slower. The release of the biphasic calcium phosphate cement system may be related to its degradation performance. COSM attached to the surface and pores of BCP will be released into the solution and enter a sudden release state. With the disintegration and degradation of BCP, the embedded COSM will be gradually released and enter a long-term sustained-release state.

### 2.4. In Vitro Cell Proliferation

The cell proliferation and toxicity test is a kind of biocompatibility test which can reflect the toxicity or growth of drugs and materials in the biological environment to a certain extent, especially in osteogenesis and angiogenesis, which play important roles in bone development and fracture healing [30].

**Figure 6:**
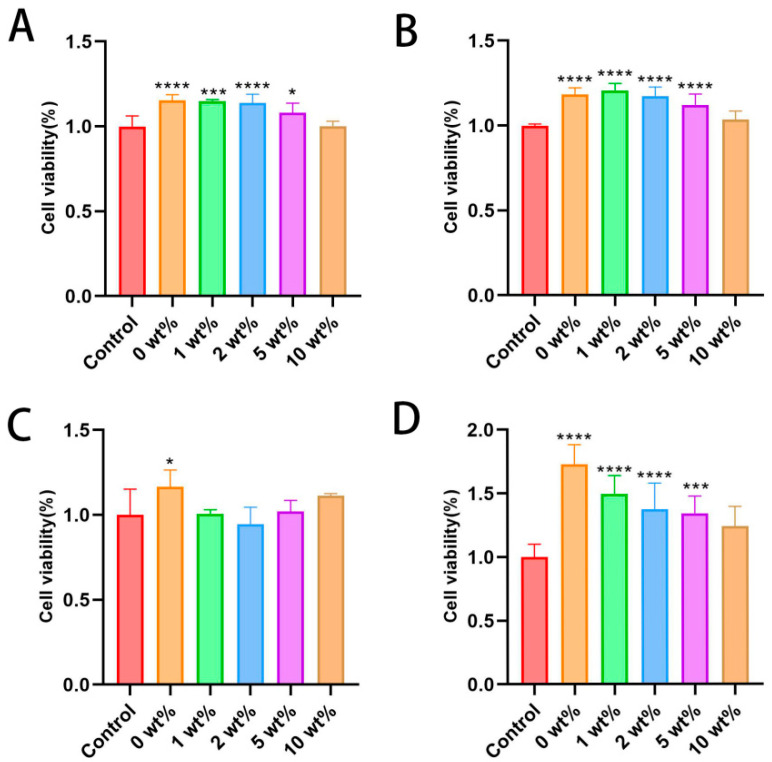
Cell proliferation in vitro. (**A**,**B**) CCK-8 method was used to determine the reproductive rate of MC3T3-E1 in different concentrations of leaching solution for 1 day (**A**) and 3 days (**B**) (*n* = 6, mean ± SD. Note: compared with control group, * *p* < 0.05, *** *p* < 0.001, **** *p* < 0.0001). (**C**,**D**) CCK-8 method was used to determine the reproductive rate of HUVEC in different concentrations of leaching solution for 1 day (**C**) and 3 days (**D**) (*n* = 6, mean ± SD. Note: compared with control group, * *p* < 0.05, *** *p* < 0.001, **** *p* < 0.0001).

Mouse osteoblast MC3T3-E1 and human umbilical vein endothelial cell HUVEC were used to evaluate the cytotoxicity of BCP bone cement granule extracts with different concentrations of COSM in normal culture medium as the control group. The data show that the cell proliferation rate of all materials at all concentrations is greater than 80% after 1 day of culture (Figure 6A). According to the ISO 10993-5-2009 standard [31], it shows that the extracts at all concentrations within 1 d have no obvious toxicity to MC3T3-E1. After 3 days of culture, compared with the normal control group, the cell proliferation of each group was further increased, and the differences in other groups were statistically significant (*p* < 0.05) except for 10 wt% COSM-BCP granule extract (Figure 6B). It shows that the bone cement extract and COSM contained in it play a positive role in cell compatibility to some extent, but a too high concentration of COSM will reduce cell proliferation.

Similarly, after HUVECs were cultured in different COSM-BCP bone cement particle extracts for 1 day, the cell proliferation rate of all materials at all concentrations is also greater than 80%, and the toxicity of extracts at different concentrations to HUVECs is first-class, with low cytotoxicity (Figure 6C). After 1 day of culture, 0 wt% COSM-BCP particles were statistically significant compared with the control group. After 3 days of culture, the cell proliferation of each group increased further, and the differences among other groups were statistically significant (*p* < 0.05) except for 10 wt% COSM-BCP granule extract (Figure 6D). It can be seen that the extracts of COSM-BCP and COSM have no obvious toxicity to HUVECs and have certain promotion effects.

### 2.5. In Vitro Cell Migration Ability

**Figure 7:**
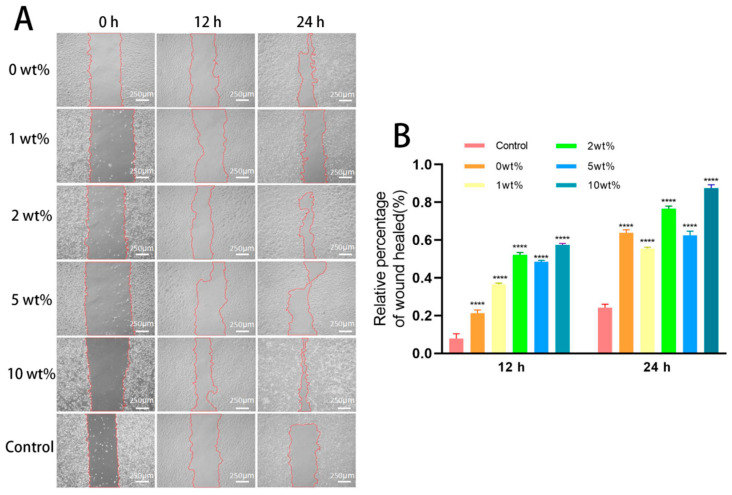
In vitro cell migration ability results. (**A**) Effects of different concentrations of extracts on migration ability of HUVEC at different time points. (**B**) Quantitative analysis of the healing rate of HUVEC at different time points in different concentrations of leaching solution environment (*n* = 3, mean ± SD. Note: compared with control group, **** *p* < 0.0001).

Cell migration is a fundamental function of normal cells and plays a crucial role in physiological processes, such as bone defect repair and wound healing. The migration of HUVECs from the scratch edge to the blank area was evaluated using a scratch test, and the migration abilities of the cells in each group were compared. The experimental results show that, with the increase in culture time, the scratch area of each group of cells decreased (Figure 7A). Compared with the control group, the COSM-BCP granule extract had no inhibitory effect on the healing rate of HUVECs after 12 h and 24 h. Compared with the control group, the differences in each group were statistically significant (*p* < 0.05). When treated with 10 wt% COSM-BCP particle extract especially, the scratch area was obviously reduced at 24 h and the healing rate was close to 80% (Figure 7B).

### 2.6. In Vitro Angiogenesis Ability Results

There is evidence that blood vessels, especially their endothelial cells (ECs), control the growth, balance, and regeneration of bones. Kusumbe and colleagues have proved that bone blood vessels contain endothelial cells that specifically support bone maturation and regeneration. Angiogenesis is a key process in the process of tissue repair and regeneration [32]. When cell proliferation occurs, ECs migrate to the tissue defect area, and the ECs will build into a tubular structure in this process [33].

**Figure 8:**
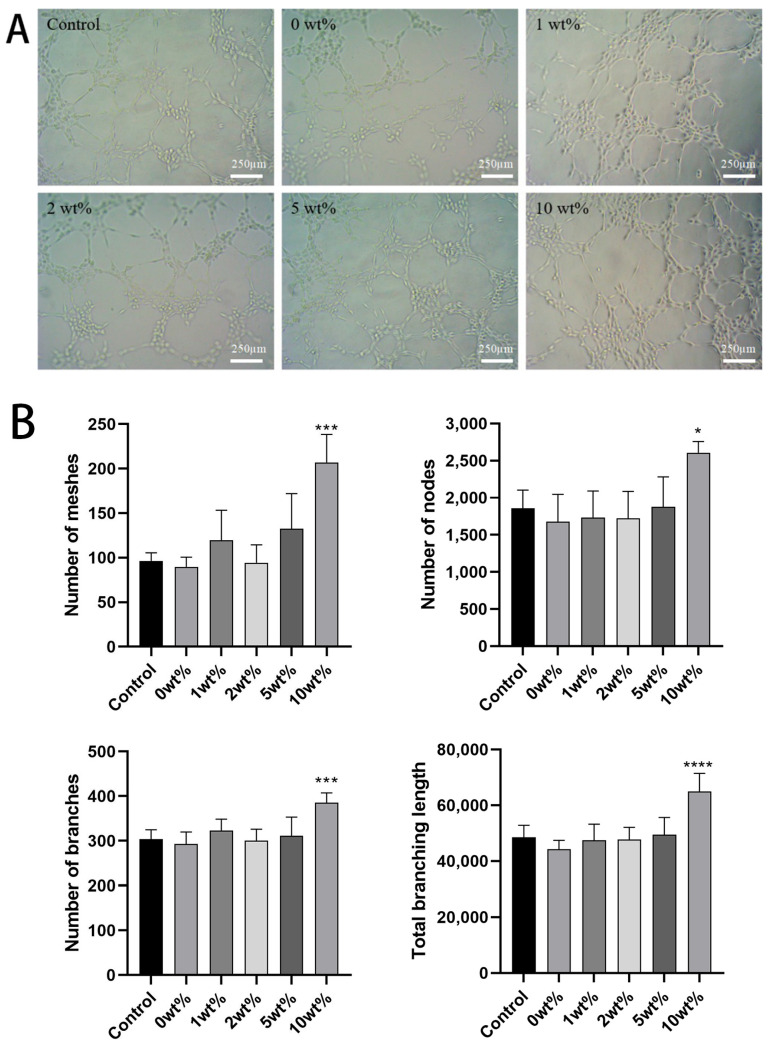
(**A**) Effect of different concentrations of leaching solution on the tube-forming ability of HUVEC at different time points. (**B**) Correlation quantitative analysis of HUVEC’s tube-forming ability at different time points in different concentrations of leaching solution environment (*n* = 5, mean ± SD. Note: compared with control group, * *p* < 0.05, *** *p* < 0.001, **** *p* < 0.0001).

The experiment of tubule formation showed that both the BCP leaching medium containing COSM and the normal group could induce HUVECs to arrange into tubule-like structures. There was no significant difference between the two groups of BCP before the addition of COSM with a concentration of 5 wt%, and the results were not statistically significant compared with those of the control group. In 10 wt% COSM-BCP, the lumen formation number, the total length, and the number of nodes were significantly different from those in the control group (*p* < 0.05). It is preliminarily proved that COSM-BCP extract can promote the tube-forming effect of HUVECs (Figure 8).

### 2.7. Expression Results of Angiogenesis-Related Genes in HUVECs

**Figure 9:**
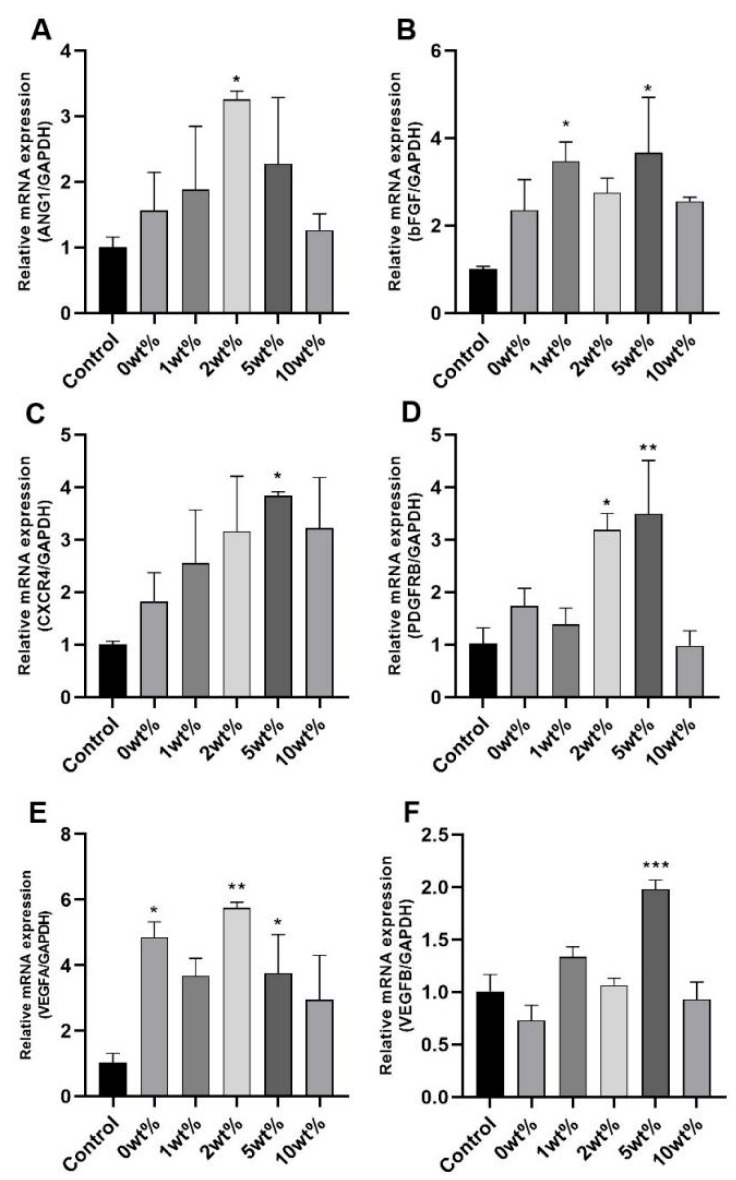
Quantitative analysis of scratch area of HUVEC cells: expression of ANG1 (**A**), bFGF (**B**), CXCR4 (**C**), PDGFB (**D**), VEGFA (**E**), and VEGFB (**F**) genes (*n* = 3, mean ± SD. Note: compared with control group, * *p* < 0.05, ** *p* < 0.01, *** *p* < 0.001).

In order to study the angiogenesis mechanism of COSM-BCP particles on HUVECs, the expression of the main regulatory factors of vascular growth was measured. In cultured HUVECs, RT-PCR analysis of cell lysis after treatment with COSM-BCP granule extract showed that they could all increase the mRNA level (Figure 9), and the expression effect of HUVECs treated with COSM-BCP granule at 5 wt% concentration was the most significant (*p* < 0.05). The results showed that COSM-BCP promoted the up-regulation of a series of cytokine parameters and promoted angiogenesis, which was consistent with the experimental results of migration and tubule formation.

## 3. Discussion

Bone defects or insufficient bone mass problems often occur due to trauma, infection, tumor resection, and fracture, which require rapid and reliable repair to restore function. Thus, bone repair is not only an important task in medical practice, it is also a complex scientific challenge. This process requires materials and technology to be able to meet a range of biological and engineering needs, including restoring the anatomical and mechanical function of bone, facilitating the generation of new bone tissue, and compatibility with the patient’s biological system and immune environment. At the same time, the individualized needs of different patients, such as the size, location of defects, and complications (such as diabetes or osteoporosis), also lead to a greater requirement for bone repair programs.

The method of producing two different calcium phosphate (α- and β-) crystals to obtain calcium phosphate has been studied; this includes obtaining α- and β- from β-TCP phosphate powder by firing prefabricated powder at a temperature above the β-TCP to α-TCP phase transition temperature [34,35]. The reaction rate of a solid-state reaction depends on the diffusion rate of atoms in the raw materials at the microlevel, and depends on the contact area, reaction temperature, and reaction time of each phase participating in the reaction at the macrolevel [14]. In order to obtain a part of α-TCP, the precursor of calcium phosphate must be calcined at a certain temperature. This requires calcination above the β→α transformation temperature. But methods for ensuring the control of this temperature and time have not been agreed upon in the literature [36,37,38,39].

This study aimed to investigate β- and α- mixed powders of tricalcium phosphate prepared from β-TCP powder. Therefore, here, we converted CaCO_3_ and CaHPO_4_ into β-TCP precursors through a high-temperature solid-phase reaction. The temperature was set at 900 °C, and the tests were conducted for 1 h, 2 h, and 3 h. The aim of this study was to identify the effect of intermediate insulation durations on synthetic β-TCP. The β-TCP synthesized from these three insulation times in an XRD pattern showed strong diffraction peaks at 2θ = 31.026°, 34.371°, 27.769°, 52.944°, 25.802°, 32.448°, 46.968°, 17.004°, etc. The high-temperature solid-phase reaction approach is a process in which a new solid product is produced by a chemical reaction between a solid and a solid. The reactants need to diffuse to the interface before the chemical reaction occurs; the reaction is controlled by the surface diffusion mechanism; the reaction rate of the solid-phase reaction depends on the microscopic diffusion rate of the atoms inside the raw material; the macroscopic diffusion rate depends on the contact area, the reaction temperature, and the reaction time. To obtain a fraction of an α-TCP, the calcium phosphate precursor must be calcined at a certain temperature, which requires calcination above the β to attain the α conversion temperature. Nurse et al. determined the phase transition temperature to be 1125 °C [40]. However, in practice, this temperature is considered to be too low for a proportional or complete conversion. Thermal analyses performed in different reports have determined that the conversion temperatures from β to α are 1115 ± 10 °C [36], 1150 °C [37,38], 1157–1195 °C [39], and 1182.4 °C [41]. The synthesis of undoped α-TCP via solid-phase reaction typically requires a calcination temperature in the range of 1300–1400 °C [14]. Therefore, based on this temperature range, we raised the temperature to 1200–1400 °C and explored it. It was found that, in the XRD pattern, 2θ = 30.753°, 34.182°, 22.902°, 22.723°, 24.098°, 30.601°, 34.604°, and 31.249°, and other positions, have strong diffraction peaks. The residence time at the calcination temperature must be sufficient to ensure the complete conversion of β-TCP to α-TCP, and this duration depends on the quantity of material being synthesized [14]. For solid-phase reactions, most authors utilize residence times between 4 and 6 h, while the shortest reported time is 1 h [42]. If there is enough time for the complete formation of alpha-TCP to occur, then extending the residence time does not appear to significantly affect the amount of alpha-TCP phase produced during synthesis [43]; this observation is consistent with the findings of our study.

As can be observed from the SEM images, the micromorphology of bone cement showed significant changes at different α-TCP/β-TCP molar ratios; the crystal structure of β-TCP is hexagonal, which is more stable than the monoclinic crystal system of α-TCP. This structural stability leads to the low solubility of β-TCP in aqueous solution, and it is not easy to release enough Ca^2+^ and PO_4_^3−^; accordingly, it cannot reach the supersaturated state in which it could form hydration products (such as CDHA). The hydration reaction requires high activation energy, and it is difficult to achieve rapid crystalline phase transformation at room temperature. With an increase in the α-TCP/β-TCP molar ratio, the hydration degree of α-TCP particles is enhanced, and the surface of α-TCP particles will quickly dissolve and release Ca^2+^ and PO_4_^3−^. With the dissolution, the concentration of Ca^2+^ and PO_4_^3−^ in solution will gradually increase. When the concentration of these ions reaches or exceeds the supersaturation threshold of CDHA, CDHA nucleation begins. In the nucleation process, calcium ions and phosphate ions in the solution continue to be consumed; they gradually form CDHA crystals and finally cover the surface of the BCP.

With the increase in COSM content, the surface structure gradually becomes more compact, and the pores are filled, which indicates that COSM participates in the hydration process of cement delays the hydration of α-TCP and makes the CDHA crystal grow more fully, thus improving the mechanical properties. The lamellar CDHA structure and dense surface help to improve the compressive strength of the product. Jan et al. found that introducing an appropriate amount of CDHA into α-TCP helps to improve the compressive strength of its composite particles [44]. The experimental results show that the compressive strength increases significantly with an increase in α-TCP content and COSM loading, which is consistent with the SEM observation results. The porosity of calcium phosphate significantly impacts its biological activity. Higher porosity enhances surface area contact with body fluids, thereby increasing the dissolution rate. Additionally, surface pores influence protein adsorption [45]. Generally speaking, when the porosity of biomaterials is 50~90%, it is conducive to cell adhesion, providing enough space for new bone tissue regeneration, allowing blood vessels and bones to grow inward, and at the same time facilitating the flow of body fluids and the transportation of nutrients [46]. In addition, the pore size also has an effect on bone degradability and bone conductivity. At the same time, pore sizes larger than 100 μm will affect the mechanical strength and shape of calcium phosphate. Excessive porosity greatly reduces the strength of materials, so a balance needs to be struck between porosity and mechanical properties [47]. In this study, although the porosity of COSM-BCP is close to the ideal value, it is still low and does not achieve the best angiogenesis effect. Therefore, it is necessary to further optimize the formulation of composite particles to enhance their related physicochemical properties in future studies.

We studied the biocompatibility and angiogenesis of COSM-BCP in vitro and found that the survival rate of COSM-BCP extracts with different concentrations on both types of cells was more than 100%, and the cells were in good condition. According to the cell migration ability, COSM-BCP granule extract had a positive effect on HUVECs. According to the results of tubule formation experiment, 10 wt% COSM-BCP has a significant effect on HUVECs. The expression of angiogenic genes was detected, and the results showed that the extract of COSM-BCP could increase the mRNA expression level and promote the up-regulation of a series of cytokines. Compared with the control group especially, the COSM-BCP particle-leaching culture medium increased the expression of several factors related to angiogenesis in HUVECs, including CD31, VEGF, and ANG. In the process of angiogenesis, VEGF regulates the recruitment, proliferation, and differentiation of endothelial progenitor cells through the VEGF-VEGFR pathway, which plays a central role in angiogenesis. Among them, CD31, as a classic angiogenic factor, is mostly distributed at the junction between endothelial cells, which actively participates in the wound-healing process and activates angiopoietin ANG. In addition, angiogenesis is regulated by many complex signal pathways, among which VEGF-VEGFR and the ANG-Tie2 axis play important roles [48]. It is found that ANG can promote or inhibit angiogenesis with or without VEGF expression. In addition, there is a certain synergistic effect between VEGF and ANG, which indicates that VEGF and Ang may jointly promote angiogenesis.

The actual bone repair efficacy and long-term safety of COSM-BCP need to be further verified by systematic animal model experiments. We can use the rat skull defect model of a rabbit femur condylar defect model to implant CPC and set bone autografts or PMMA as the control. The bone metabolism and repair processes of rats are similar to those of humans, especially in the early stages of bone defect repair. Therefore, the rat skull defect model can better simulate the repair process of human bone defects. Silva et al. [49] compared the effect of the demineralized bone matrix and calcium phosphate bone cement on bone regeneration in the rat skull defect model. The results showed that calcium phosphate bone cement showed good effects in promoting new bone formation. Alonso et al. [50] studied the bone repair effect of 3D-printed polylactic acid–bioceramic calcium phosphate (PLA-bioCaP) composite material in a rabbit femoral condyle defect model. It was found that the composite material could significantly promote the healing of bone defects, showing good biocompatibility and osteogenic ability. The experimental design should involve multiple time points, such as 4, 8, 12, and 24 weeks. Comprehensive analysis should be performed in combination with HE staining, Masson staining, Micro-CT bone volume fraction analysis, and biomechanical tests; additionally, the long-term safety of the immune microenvironment should be evaluated by detecting inflammatory factors (such as IL-6, TNF-α) and the degree of immune cell infiltration. Before we undertake this experiment, we need to explore related aspects, such as the impact of the material on long-term toxicity in vivo, the evaluation of therapeutic effects of different models, the elimination mechanism of degradation products in vivo, the impact of degradation products on liver and kidney function, and the standardized production of the material before clinical use to ensure the consistency of CPC quality and performance. In future work, we will explore the situation of calcium phosphate bone cement in the bone repair of different body parts through models such as the rabbit femoral defect model and the rat skull defects model. During this process, the materials will be continuously optimized. We may consider incorporating trace elements such as strontium, magnesium, and zinc or complex growth factors to enhance the biological activity of the materials. Technically, porous CPC scaffolds can be prepared by combining 3D printing technologies to simulate the natural trabecular bone structure. Gene therapy can also be introduced, using CPC as a gene vector to continuously release bone-regeneration-promoting genes (such as VEGF, BMPs) and activate the endogenous repair mechanism. For the expansion of interdisciplinary applications, the injection performance of CPC can be optimized for immediate alveolar socket filling or peri-implant bone augmentation, or the porous structure of CPC can be used as a nerve catheter scaffold; this could be combined with neurotrophic factors to promote nerve regeneration.

In this study, although the correlation between the pathways involved in COSM and the upstream and downstream targets at the molecular level has not been clarified, and its angiogenic activity has only been verified in vitro, it has been proved that COSM has a promoting effect on angiogenesis; furthermore, we have shown that it can significantly improve the migration and tubular structure formation ability of HUVECs in terms of gene expression. It has been proved that α/β-TCP biphase bone cement prepared in a high-temperature solid-phase reaction has controllable physical properties. These findings not only provide a new perspective for the application of chito-oligosaccharides in bone repair but also provide a scientific basis for the further development and optimization of biphasic bone cement.

## 4. Materials and Methods

### 4.1. Materials

Calcium carbonate (CaCO_3_), anhydrous calcium hydrogen phosphate (CaHPO_4_), anhydrous citric acid (C_6_H_8_O_7_), disodium hydrogen phosphate anhydrous (Na_2_HPO_4_), and 3,5-dinitrosalicylic acid were purchased from MACKLIN Biochemical Technology Co., Ltd. (Shanghai, China). Glucosamine was purchased from Shanghai Aladdin Biochemical Technology Co., Ltd. (Shanghai, China). PBS, DMEM high glucose medium, CCK-8, and DEPC were purchased from Dalian Meilun Biotechnology Co., Ltd. (Dalian, China). Matrix-Gel matrix glue was purchased from BiYuntian Biotechnology Co., Ltd. (Linfen, China). Fetal calf serum (FBS) was purchased from Prossay Life Technology Co., Ltd. (Wuhan, China). DMSO (cell culture grade) was purchased from Sigma-Aldrich (St. Louis, MO, USA).

### 4.2. Preparation of Biphasic Calcium Phosphate Cement Loaded with COSM

#### 4.2.1. Preparation of Biphasic Calcium Phosphate (BCP) Powder

In this study, α-TCP and β-TCP powders with different proportions were prepared using a high-temperature solid-state method. Calcium hydrogen phosphate and calcium carbonate were evenly mixed in the molar ratio of 2:1 and sintered at 900 °C for 1 h, 2 h, and 3 h; the powder passes through a 100-mesh screen after cooling. The powder was calcined at 1200 °C, 1300 °C, and 1400 °C for 2 h, 3 h, and 4 h; then, it was quenched rapidly in the air and ground in a planetary ball mill (Chishun Development Technology Company, Nanjing, China) until it passed through a 325-mesh screen. Thus, biphasic calcium phosphate powder with a particle size of about 50 μm was obtained.

#### 4.2.2. Preparation of Bone Cement Pastes

A measure of 1.0 g of BCP powder was mixed with phosphoric acid buffer solution at a solid–liquid ratio of 1:0.5 (L/Q, g/mL), filled into a Φ 6 mm × 12 mm cylindrical hole mold for compaction, and then the bone cement particles were pushed out after molding. The bone cement particles were cured in a constant temperature box at 37 °C and 100% humidity. After 3 days, the compressive strength and phase analysis could be tested. The curing solution contained 2.09 wt% citric acid monohydrate and 5 wt% disodium hydrogen phosphate.

#### 4.2.3. Chitosan Loading

Different amounts of COSM (based on the study of COSs, we named COSs with MW ≤ 3000 Da as COSM) were dissolved in a curing solution based on 2.09% monohydrate citric acid and 5% disodium hydrogen phosphate, wherein that mass concentration of chitosan oligosaccharide in the solution is 0–10 wt%. COSM was determined by 3, 5-dinitrosalicylic acid method (DNS method: the principle of DNS can be seen in the Supplementary Materials Dataset), and the sample solution was determined by an MAXM4 microplate reader (Yiheng Science and Technology Company, Qinhuangdao, China). The successful loading of COSM was indirectly determined in the wavelength range of 540 nm.

### 4.3. Physical and Chemical Properties Analysis

#### 4.3.1. Characterization of BCP

The phase composition of BCP powder was analyzed by a MiniFlex 600 X-ray diffractometer (XRD, Rigaku Company, Tokyo, Japan), and a quantitative analysis was performed using the RIR method. The calculation formula of the RIR method is as follows:

(2) $$w_{i}=\frac{I_{i}∕S_{i}}{\sum \left(I_{j}∕S_{j}\right)}$$

A Zeiss Sigma300 field emission scanning electron microscope (SEM, Carl Zeiss Company, Oberkochen, Germany) was used to observe the micromorphology of the biphasic calcium phosphate powder and the micro-results of the samples after the calcium phosphate cement was cured. The porosity, pore size, and pore distribution of the product were measured using an American McMuritic AutoPore IV 9520 mercury porosimeter (Micromeritics Instrument Corporation, Norcross, GA, USA).

#### 4.3.2. Determination of Curing Time and Compressive Strength

The curing time was determined by the Vicat needle method, and the initial setting time and final setting time of the bone cement were used. The curing time was subject to the final setting time. The mixed mud was put into the mold and tested using a Vicat needle every 0.5 min until the bone cement was completely solidified. The test samples were removed and dried; then, the upper and lower surfaces of the cylindrical sample were polished with sandpaper. An HZ-1004 universal mechanical testing machine (Hengzhun Instrument Technology Company, Shanghai, China) was used to carry out mechanical testing on the solidified sample, with a test loading rate of 0.5 mm/min.

#### 4.3.3. In Vitro Release of COSM

The DNS method established above was used to detect the drug concentration released by COSM from the sample in vitro. The drug-release sample was a cylinder with a diameter of 6 mm and a height of 12 mm, and PBS (pH = 7.4) was added as the drug-release medium according to the ratio of 10 mL/g. At selected time points (3, 6, 12, 18, 24, 48, 72, 96, 120, 144, 168 h), the liquid was taken and stored for testing, and the same amount of PBS solution was added to the original drug release system. The obtained drug solution was treated by the DNS method and tested according to the established equation. The equations can be accessed in Figures S2–S4 of the Supplementary Materials Dataset.

### 4.4. In Vitro Cell Culture Study

#### 4.4.1. Preliminary Study on the Toxicity of Osteoblasts and Vascular Cells

The cell lines were purchased from the cell bank of China Academy of Sciences. Mouse osteoblast MC3T3-E1 mobile line and human umbilical vein endothelial cell HUVEC mobile line were used. The cells were cultured in DMEM medium containing 10% fetal bovine serum and 1% fetal bovine serum in a humidified incubator containing a 5% carbon dioxide atmosphere.

In this study, the CCK-8 method was used to determine the cell survival rate to evaluate the biocompatibility of COSM-BCP bone cement particle extract with different concentrations. Human normal MC3T3-E1s and HUVECs were cultured in vitro, and the cell state was observed using a microscope according to 8000–10,000 cell plates per well in 96-well plates. After about 10–12 h of culture, 0, 1, 2, 5, and 10 wt% groups of COSM-BCP granules were set according to the experimental needs, and each group was set with 6 holes, and the cells were cultured for 1 d and 3 d, respectively. The cell proliferation of each group was tested using a CCK-8 kit.

#### 4.4.2. Cell Scratch Test In Vitro

The cell migration experiment was carried out as described above [48]. HUVECs were seeded on a 6-well plate at a density of 1 × 10^6^ cells/well. After growing to 85–90% fusion, the cells were scraped with the tip of sterile disposable pipette to form scratches. Then, the cells were washed twice with PBS, and the cells were incubated with different concentrations of COSM-BCP extracts (BCP containing 0 wt%, 1 wt%, 2 wt%, 5 wt%, and 10 wt% of COSM), with a blank culture medium used as the blank control group (none contained fetal bovine serum); each group was provided with three re-wells. After incubation for 12 h and 24 h, the wound area was photographed using an inverted phase contrast microscope (Olympus, Tokyo, Japan), and the migration area was calculated by ImageJ 1.52a software to analyze the migration of the cells.

#### 4.4.3. In Vitro Tubule Formation Experiment

HUVECS (6 × 10^3^ cells/well) were inoculated into a 48-well culture plate, which was precoated with Matrix-Gel matrix adhesive (CAT # C0372-5; Blue Sky Biotechnology Company, Dalian, China). After incubation at 37 °C for 4 h, we used ImageJ software to quantify the characteristics of the pseudo-capillary network in HUVECs. We used the inverted phase contrast microscope to calculate the number of tube branches under the magnification of ×10, measured the length (pixel) of the tube, and calculated the number of capillary network grids, nodes, and branches of each culture plate in three random fields [51,52].

#### 4.4.4. RNA Isolation and Real-Time PCR

The primer genes were identified through a review of the relevant literature and an analysis of the published data. All primers were synthesized by Shanghai Shenggong Biological Co., Ltd. (Shanghai, China), and their sequences are provided in Table 1 [53,54].

Total RNA was extracted using TRIzol (Takara, Tokyo, Japan) following the manufacturer’s protocol. The RNA concentration was measured using an ultra-micro UV spectrophotometer. cDNA was synthesized after the removal of genomic DNA through reverse transcription. Synthetic primers for reverse transcription cDNA, along with reagents from the TaKaRa TB Green Premix Ex Taq™ II kit (including upstream primers, downstream primers, TB Green Premix Ex Taq™ II, cDNA, and DEPC water, Takara, San Jose, CA, USA), were added to a Roche 96-well PCR plate (Roche Holding AG, Basel, Switzerland) in the specified amounts and centrifuged for 2 min. A fluorescence quantitative polymerase chain reaction (qPCR) was conducted using a PCR instrument, and data were collected following the amplification process. Relative gene expression levels were calculated using the 2^−ΔΔCt^ method.

### 4.5. Statistical Analysis

The data obtained in this experiment were analyzed and plotted by SPSS 20.0 and GraphPad Prism 8.3; the final results from the data were expressed in the form of mean SD. The data were compared using one-way ANOVA, with *p* < 0.05 indicting statistical significance.

## 5. Conclusions

In this study, we developed a biphasic calcium phosphate (BCP) bone cement carrier which is suitable for clinical operation; this was loaded with a chitosan oligosaccharides to produce a COSM drug with an angiogenesis effect, obtaining COSM-BCP bone cement particles. Biphasic tricalcium phosphate with an α-phase/β-phase molar ratio of about 1:1 was obtained using the high-temperature solid-state method; this involved calcination at 900 °C for 1 h, followed by calcination at 1300 °C for 2 h. The curing time was 26.5 1 min; the compressive strength was 27.98 2.66 MPa; the porosity was 50.18%. These values match the pore characteristics of normal human bones. COSM was released suddenly and then slowly in vitro, and it was released stably and continuously in the drug-loaded system. The proliferation efficiency of COSM-BCP on MC3T3-E1s and HUVECs was over 100%, and the cell state was good. It was found to have a positive effect on the migration ability and tubule formation of HUVECs cells. Furthermore, it was found to promote up-regulation in a series of cell angiogenic factors. To sum up, BCP bone cement is made of non-toxic, harmless inorganic materials; it has certain advantages, such as a stable preparation process and good reproducibility, and it is a good drug-loading system for COSM. In addition, it has good biocompatibility and vasculogenic activity, and has great potential medical value; therefore, this study and its findings provide an important scientific research foundation for clinical applications.

## Figures and Tables

**Table 1:** The sequence of the primers for RT-PCR.

Gene Name	Primer Sequence (5′→3′)
GAPDH	F: GAAAGCCTGCCGGTGACTAA
	R: GCCCAATACGACCAAATCAGAG
ANG1	F: CAGTGGCTGCAAAAACTTGAGA
	R: AGTCTGAGAGAGGAGGCTGG
bFGF	F: GCGACCCTCACATCAAGCTA
	R: AGCCAGGTAACGGTTAGCAC
CXCR4	F: GGGCAGAGGAGTTAGCCAAG
	R: GGGCTAAGGGCACAAGAGAA
PDGFRB	F: CCATCAGCAGCAAGGCGA
	R: AGCAGGTCAGAACGAAGGTG
VEGFA	F: ACAAATGTGAATGCAGACCAAA
	R: ACCAACGTACACGCTCCAG
VEGFB	F: CAAGTCCGGATGCAGATCCT
	R: TCTGCATTCACACTGGCTGT

## Data Availability

The datasets generated and/or analyzed during the current study are available from the corresponding authors on reasonable request.

## Supplementary Materials

The following supporting information can be downloaded at: https://www.mdpi.com/article/10.3390/molecules30112286/s1, Figure S1: Reaction principle of DNS method; Figure S2: The absorption spectra (A) and partial enlargement (B) of Water-DNS+Glu and Water-DNS; Figure S3: The absorption spectrum of Water-DNS+Glu at 400-700 nm; Figure S4: Linear fitting curve with glucosamine hydrochloride as standard; Figure S5: The XRD pattern reproduced by the a-TCP:b-TCP ratio; Figure S6: XRD analysis of particles after hydration; Figure S7: XRD analysis of the complete hydration of α-TCP to form CDHA. Refs. [55,56] are cited in the Supplementary Materials.

## Abbreviations

Abbreviation	Full Name
α-TCP	α-tricalcium phosphate
ACP	Amorphous calcium phosphate
ADSCs	Adipose-derived stem cells
ALP	Alkaline Phosphatase
β-TCP	β-tricalcium phosphate
bFGF	Fibroblast Growth Factor
BMP	Bone Morphogenetic Protein
BCP	Biphasic calcium phosphate
CD31	Platelet endothelial cell adhesion molecule-1
CaP	Calcium phosphate
CPP	Calcium pyrophosphate
CBD	Cannabidiol
CPC	Calcium phosphate cement
CDHA	Calcium-deficient hydroxyapatite
COSM	COS (MW ≤ 3000)
CCK-8	Cell embryo osteoblast precursor cells
DCPA	Dicyclopentenyl acrylate
DNS	3,5-dinitrosalicylic acid
DMSO	Dimethyl sulfoxide
ECM	Extracellular matrix
Emcm	Endomucin
EC	Endothelial cell
ERK	Extracellular regulated protein kinases
FBS	Fetal bovine serum
Glu	Glucosamine hydrochloride
HAP/HA	Hydroxyapatite
HIF-1α	Hypoxia inducible factor 1 subunit alpha Gene
HUVECs	Human Umbilical Vein Endothelial Cells
IL-6	Interleukin-6
IL-1β	Interleukin-1β
MAPK	Mitogen-activated protein kinase
MIP	Mercury intrusion porosimetry
MC3T3-E1s	Mouse embryonic osteoblast precursor cells
MCP-1	Monocyte chemoattractant protein-1
MSC	Mesenchymal stem cells
OPG	Osteoclastogenesis inhibitory factor
PBS	Phosphate Buffer system
PCR	Polymerase Chain Reaction
PDGF	Platelet-derived growth factor
PLA	Polylactic acid
PGA	Polyglycolic acid
PLGA	Poly(lactic-co-glycolic acid
PMMA	Polymethyl Methacrylate
PEEK	Poly(ether-ether-ketone)
RANKL	Receptor Activator of Nuclear Factor-κ B Ligand
SLIT3	Slit guidance ligand 3 Gene
SEM	Scanning electron microscope
TNF-α	Tumor necrosis factor-α
TGF-β	Transforming Growth Factor-β
TTCP	Tetracalcium phosphate
VEGF	Vascular endothelial growth factor
XRD	X-ray Diffraction
