# Supplementary material for: Chitosan-Oligosaccharide-Bearing Biphasic Calcium Phosphate Bone Cement: Preparation and Angiogenic Activity In Vitro
Source: Molecules. 2025 May 23;30(11):2286. doi: 10.3390/molecules30112286 (PMC12155841; doi:10.3390/molecules30112286)
Supplement: Supplementary file 1 [file molecules-30-02286-s001.zip › molecules-3500430-supplementary.pdf]

Preparation of biphasic calcium phosphate bone cement bearing chitosan oligosaccharide and its angiogenic activity in vitro

Jianshen Liu [1,2,†], Xinghua Guo [1,2,†], Qishi Che [3], Zhengquan Su [1,2,*]

1 Guangdong Engineering Research Center of Natural Products and New Drugs, Guangdong Provincial University Engineering Technology Research Center of Natural Products and Drugs, Guangdong Pharmaceutical University, Guangzhou (510006), China

2 Guangdong Metabolic Disease Research Center of Integrated Chinese and Western Medicine, Key Laboratory of Glucolipid Metabolic Disorder, Ministry of Education of China, Guangdong TCM Key Laboratory for Metabolic Diseases, Guangdong Pharmaceutical University, Guangzhou (510006), China

3 Guangzhou Rainhome Pharm & Tech Co., Ltd，Science City, Guangzhou (510663), China

* Correspondence: suzhq@scnu.edu.cn (Z.S.); Tel.: +86-020-39352345 (Z.S.)

† These authors contributed equally to this work.

S1. Materials and methods

S1.1 Physical and chemical properties analysis

S1.1.1 Chitosan loading

Chitosan oligosaccharides are alkaline amino oligosaccharides composed of 2-amino-2-deoxy-D-glucose (glucosamine) units linked by β-1,4-glycosidic bonds, with some N-acetylglucosamine residues present. Currently, several methods are available for determining the content of chitosan oligosaccharides, including potassium permanganate titration, the phenol-sulfuric acid method, the Elson-Morgan method (acetylacetone method), the 3,5-dinitrosalicylic acid (DNS) method, and high-performance liquid chromatography (HPLC). Among these, the phenol-sulfuric acid method, Elson-Morgan method, and DNS method are indirect methods that measure reducing sugars. In 2011, Li Kecheng et al. [1] reported that the Elson-Morgan method exhibited a low recovery rate, making it unsuitable for the determination of chitosan oligosaccharide content. The phenol-sulfuric acid method demonstrated a good standard curve for glucosamine in the range of 200–3000 μg, but it tended to overestimate the chitosan oligosaccharide content. In contrast, the DNS method yielded results that closely matched theoretical values. Furthermore, numerous studies have confirmed that the DNS method exhibits excellent stability, reproducibility, high precision, and ease of operation, making it a fast and reliable approach for determining chitosan oligosaccharide content.

The principle of the DNS method is based on the reaction of 3,5-dinitrosalicylic acid (DNS) with reducing sugars produced by polysaccharide hydrolysis under neutral or slightly alkaline conditions. The DNS reagent is reduced to a reddish-brown amino compound, 3-amino-5-nitrosalicylic acid, upon heating (as shown in Fig.S1). Within a certain range, the amount of reducing sugars is positively correlated with the color intensity of the reaction solution. However, different studies have reported varying detection wavelengths for DNS, including 540 nm, 520 nm, and 490 nm, leading to

inconsistencies in the established standard curves, linear ranges, accuracy, and precision. Therefore, in this study, we established and validated a methodological system to evaluate the application of the DNS method for the determination of COSM content.

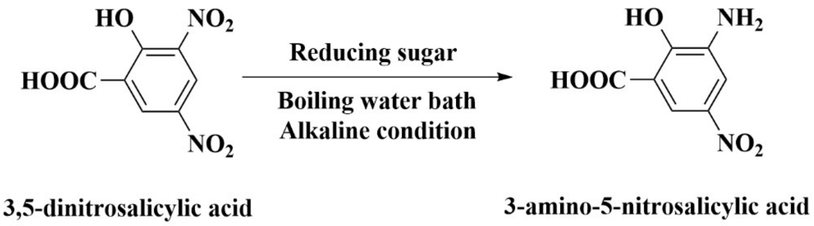

Figure S1. Reaction principle of DNS method

S1.1.2 In vitro release of COSM

To determine the optimal determination wavelength for the reaction in DNS reagent, the absorption spectra of water-DNS + Glu and Water-DNS were compared with purified water and glucosamine hydrochloride solution as controls, respectively, and the results are shown in Fig.S2. As can be seen from FIGS. Fig.S2 (A) and Fig.S3, although the absorption spectrum of the two groups reaches the maximum absorption difference at 480-490 nm, the DNS reagent itself still has a high absorption in this band, which will also interfere with the detection results of the sample. According to the principle of "maximum absorption, minimum interference", the wavelength with the minimum DNS background and higher absorption of DNS + Glu are appropriately selected as the optimal absorption wavelength, as shown in Fig.S2(B). Therefore, 540 nm is selected as the detection wavelength for determining the amount of reducing sugar content[2]. This is consistent with the absorption wavelengths selected in most studies.

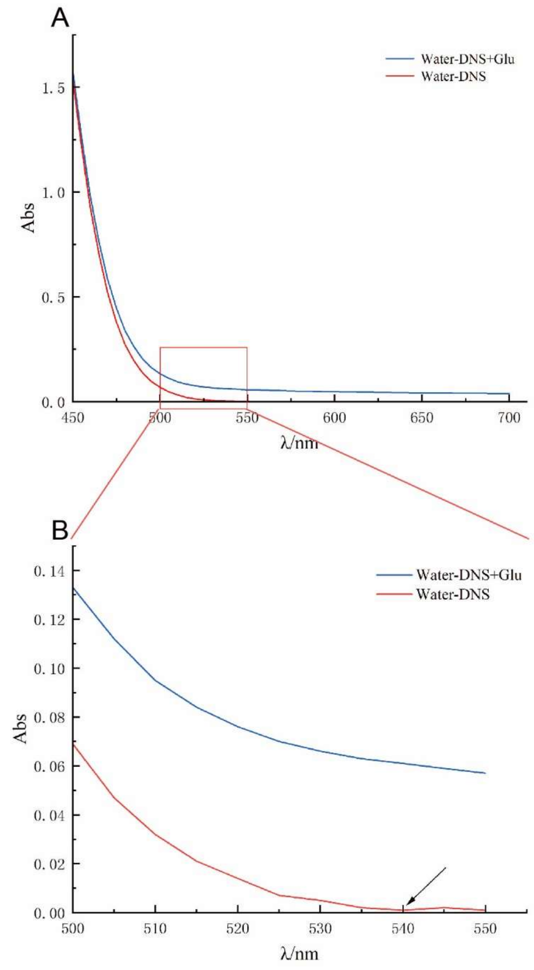

Figure S2. The absorption spectra (A) and partial enlargement (B) of Water-DNS+Glu and Water-DNS

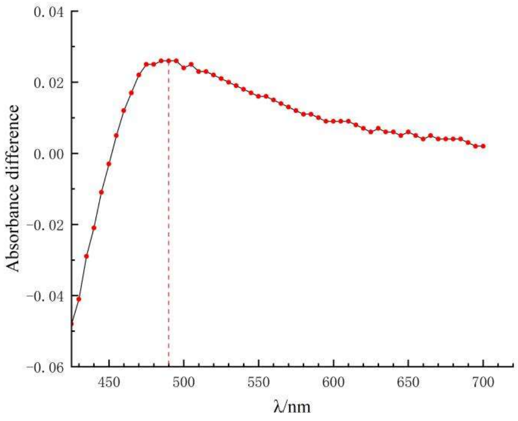

Figure S3. The absorption spectrum of Water-DNS+Glu at 400-700 nm

According to the linear range of Fig.S4, when the concentration of Glucosamine hydrochloride solution is within 100-900 μ g / mL (R2=0.9968), the standard fit curve between Glu concentration and specificity absorbance is y=0.0008x+0.0268.

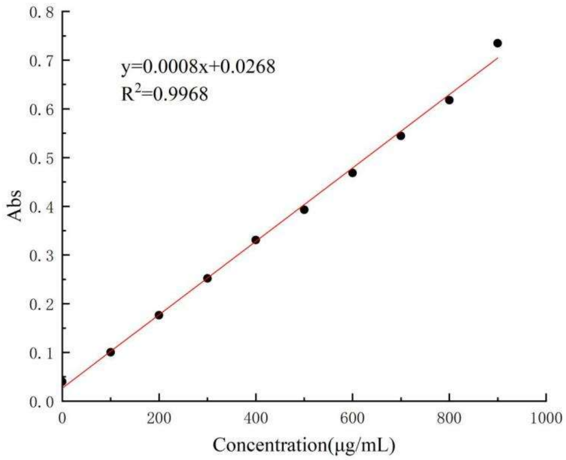

Figure S4. Linear fitting curve with glucosamine hydrochloride as standard.

S2. Results

S2.1. Material properties

S2.1.1. XRD patterns

The ratio of a-TCP:b-TCP can be stably reproduced, as shown in Fig S5.

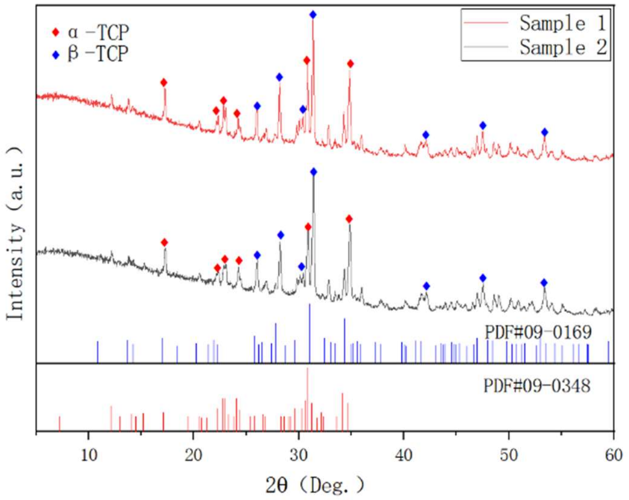

Figure S5. The XRD pattern reproduced by the a-TCP:b-TCP ratio.

Through the XRD data analysis after the hydration of the particles, it can be found that there is a peak shape of CDHA in the XRD data. Therefore, it can be confirmed that the nanocroneedle-like substance is CDHA, and β-TCP does not participate in the hydration.（Fig.S6）

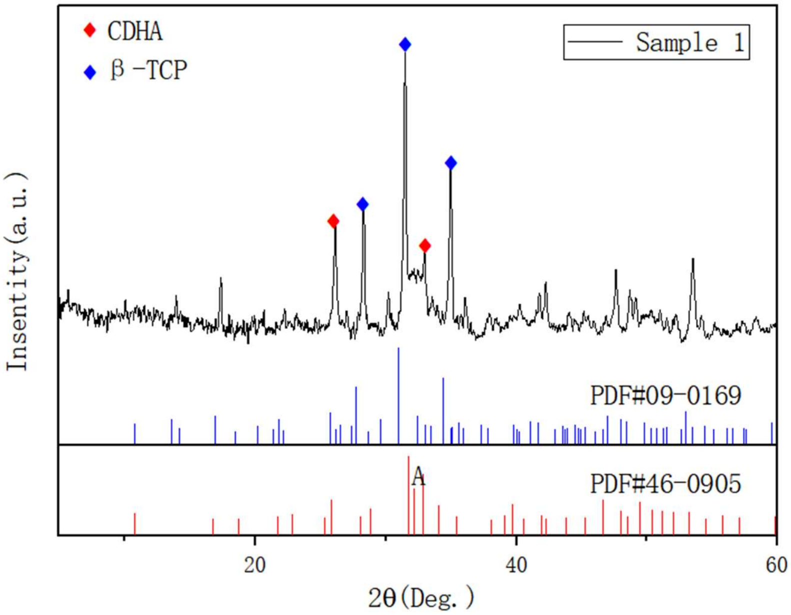

Figure S6. XRD analysis of particles after hydration.

As shown in Fig.S7, by preparing pure α-TCP into particles and then conducting hydration, XRD analysis of the hydration product revealed that α-TCP could be completely converted into CDHA. During this process, no unreacted components or other impurities were produced.

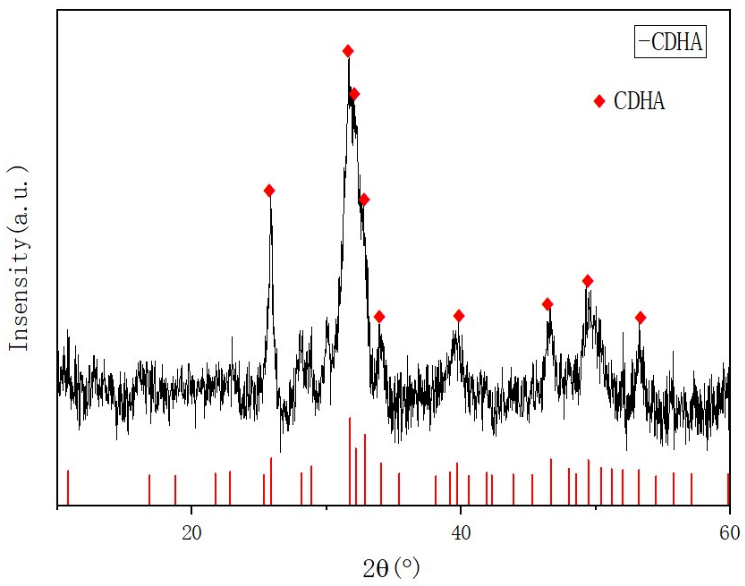

Figure S7. XRD analysis of the complete hydration of α-TCP to form CDHA.

Reference

[1] LI K-C, LI P-C, XING R-E, et al. Comparison of Determination Methods for Chitosan Oligosaccharide Content[J]. Chinese Journal of Pharmaceutical Analysis, 2011, 31(08): 1530-1532.

[2] HONG-YING W, SRGULENG Q, QIAN-CHENG Z, et al. Determination of Polysaccharide in Ophiopogon japonicus with 3,5-Dinitrosalicylic Acid Colorimeitry[J]. Journal of Shenyang Agricultural University, 2005.
